# A Simple Experiment to Test the Toxicity of Toothpaste (3T Experiment): An Observational Pilot Study

**DOI:** 10.7759/cureus.68978

**Published:** 2024-09-09

**Authors:** Thomas Mathew, Shagun Bhardwaj, Surabhi Garg, Sindhu V Nambiar, Talakad N Sathyaprabha

**Affiliations:** 1 Neurology, St. John's National Academy of Health Sciences, Bengaluru, IND; 2 Neurophysiology, National Institute of Mental Health and Neurosciences, Bengaluru, IND

**Keywords:** epipremnum aureum, money plant, public health, toothpaste, toxicity

## Abstract

Introduction

Ingredients in toothpaste can impact living cells and organisms. Fluorides in toothpaste are known to cause various disorders in both animals and humans. Based on these observations, we evaluated the effect of toothpaste ingredients on the survival of money plants (*Epipremnum aureum*).

Methodology

We selected four money plants and placed each one in a glass of water. After a three-day stabilization period, we added 100 mg of three commonly used toothpaste brands to the water of three glasses, each containing one money plant, for two weeks. One glass was maintained as a control without any toothpaste. We then observed the changes in the plants over the next four weeks.

Results

The plants exposed to toothpaste began to show discoloration within two weeks. The leaves withered and dried in all the treated plants within 30 days. In contrast, the control plant remained healthy and sprouted a new bud.

Conclusion

Our observations indicate that all three commonly used toothpaste brands were toxic to the money plant. These findings may encourage further experiments to study the toxicity of toothpaste ingredients.

## Introduction

Ingredients in toothpaste can impact living cells and organisms. Substances in toothpastes like fluoride inhibit sodium-potassium ATPase and have been implicated in various neuropsychiatric diseases [1]. Sodium lauryl sulfate (SLS), another ingredient in toothpaste, has been associated with injuries to the oral mucosa and the development of oral ulcers [2]. Heil et al. conducted a study to evaluate the DNA-damaging properties of dental materials and found that most tested materials yielded positive results in at least one genotoxic test [3]. We have also observed patients experiencing headaches, vertigo, and trigeminal neuralgia due to ingredients in toothpaste, and we have recently presented and published various case series on toothpaste-induced neurological disorders [4-8].

Given these observations, we hypothesized that toothpaste may contain toxins that can harm human cells. This prompted us to explore different models to test our hypothesis. Considering the ethical concerns and challenges associated with toxicological experimentation on animals and humans, we decided to begin our research with a pilot study involving plant experiments.

We chose the money plant (*Epipremnum aureum*) for our experiment due to its high survival capacity and resilience. These plants require only water and sunlight for survival and can thrive in nutrient-deficient environments for extended periods. The objective of this study is to evaluate the phytotoxic effects of the ingredients in three commonly used toothpaste on the survival and growth of the money plant (*Epipremnum aureum*). We hypothesize that exposure to toothpaste ingredients will have a detrimental effect on the growth and survival of money plants, leading to observable signs of phytotoxicity.

## Materials and methods

We chose three commonly used toothpaste, water-filled glass pots, and money plants (*Epipremnum aureum*) for the experiment. Equal-sized money plants, 12 cm in height with a healthy stem and two green leaves, were put in four half-water-filled, clear glass pots containing 100 ml of chlorine-free filtered water. These plants were kept at room temperature at 26°-27°C with an average humidity of 70%. All the pots were kept near the window, where they received diffuse, scattered, and bright light evenly, without direct sun exposure. These conditions were maintained constant throughout the experiment. The toothpaste was measured by an Essae AX 620 (Karnataka, India) with an accuracy of 0.001 g and a pan size of 110 mm in diameter. 

Pot 1 was the control, and pots 2, 3, and 4 were used for intervention. We added half the usual amount of toothpaste used for brushing teeth (100 milligrams) from toothpaste 1, toothpaste 2, and toothpaste 3 into pots 2, 3, and 4, respectively. The ingredients of the three toothpastes are given in Table 1.

**Table 1:** Ingredients of toothpastes used

INGREDIENTS	TOOTHPASTE 1	TOOTHPASTE 2	TOOTHPASTE 3
	Maricha(Piper nigrum) Pippali (Piper longum) Shunthi (Zingiber officinale) Tomar (Zanthoxylum armatum) Lavanga (Syzygium aromaticum) Karpura (Cinnamomum camphora) Pudina (Mentha species) Gairic powder Sodium Saccharin Sodium Benzoate	Hydrated Silica Titanium Dioxide Sodium Monofluorophosphate Calcium carbonate Sodium Lauryl Sulphate Flavour Potassium Nitrate Sodium Bicarbonate Sodium Saccharin Sorbitol Limonene Sodium Silicate Benzyl Alcohol Carrageenan Arginine	Precipitated Silica Titanium Dioxide Sodium Fluoride AC 1131 Flavour Sodium Saccharin Sorbitol Sodium Propyl Hydroxybenzoate Sodium Methyl Hydroxybenzoate Sodium Methyl Cocoyl Taurate Xanthan Gum Glycerol Strontium Acetate

We had a three-day screening period (day 1 to day 3) in which the plants were observed for their growth under normal conditions and adjustments in the set-up before the experimental phase. On day four, pot 1 was kept as a control, and toothpaste was added to pots 2, 3, and 4 between 9 and 10 AM into their respective glasses, and photos were taken each day to document the changes in the plants. We introduced 100 mg of toothpaste directly into the water without dilution. In the pre-experimental phase, we found that if we stirred the toothpaste, the color of the water became cloudy, preventing further observation of the stem of the plant. This observation made us decide not to stir in the experiment, and hence we allowed the toothpaste to dissolve naturally. The toothpastes were added daily for 14 days during the intervention period (day 4 to day 17). The water was not changed periodically, as it would have affected the distribution of ingredients and their impact on the plant. The plants were observed daily for leaf discoloration, wilting, and growth. Photographs were taken using a smartphone camera daily. Observational statistical methods were employed to analyze data. 

## Results

**Figure 1:**
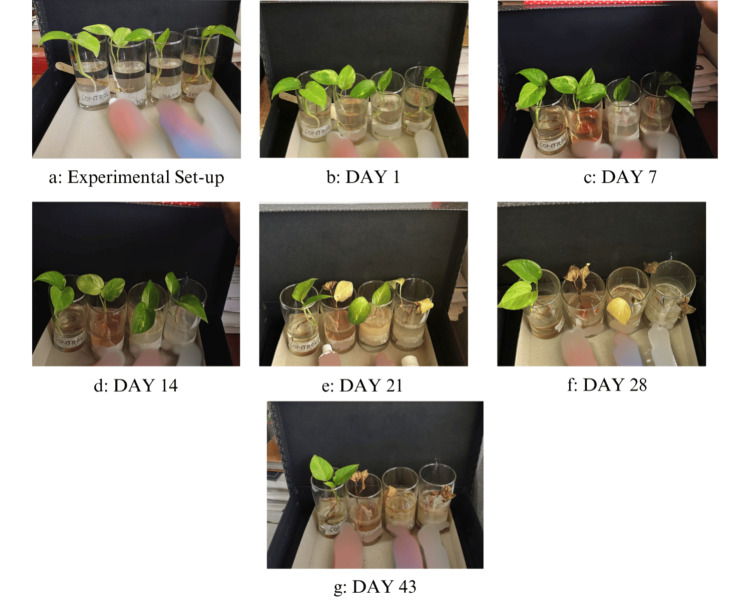
Images showing the experimental setup (left to right: Control, Toothpaste 1, Toothpaste 2, Toothpaste 3); a: Experimental set-up before the toothpastes were added; b—c: Plants in the first week of the experimental phase; d: discoloration first noted in glass 2; e: discoloration seen in glass 4; f: discoloration and withering in all glasses, control plant remained healthy; g: latest image of plants on day 43 with a new leaf in the control

During the first three-day screening period, no changes were noted in the plants. In the first 13 days of the intervention period, there were no visual changes noted in the plants. On the 14th day of the intervention, we observed yellowish discoloration of the leaves in glass 2 (toothpaste 1) (Figure 1d), on day 17, we noted similar changes in glass 4 (toothpaste 3), and on day 22, glass 3 (toothpaste 2) (Figure 1e). The leaves and the stem of the plants in glasses 2 and 4 dried up completely on day 24. The leaf and stem of the plant in glass 3 were dried on day 30. (Figure 1f). There was no wilting or new bud growth seen in the toothpaste-exposed plants. The plant in the control condition (glass 1) was healthy with new bud growth without any discoloration, wilting, or withering. (Figure 1g).

## Discussion

In the current experiment, we observed that the commonly used toothpastes were toxic to the survival and growth of money plants (*Epipremnum aureum*). This toxicity was observed between 2-4 weeks after an intervention period of two weeks. Money plants by nature have a high survival capacity and are resilient, as they require just water and sunlight for survival, and these properties have led to their use in phytoremediation [9]. The plant can not only adapt excellently to diverse climatic conditions but also be used as an accumulator of trace metals such as fluoride. Tang and Chai demonstrated the plant’s ability to extract crude oil contaminants from soil [10]. Similarly, in experiments done by Singh et al., the potential use of *E. aureum* as a phytoextractor of heavy metals was discussed, and the preliminary results revealed that *E. aureum* survives in a nutrient-deficient environment for a period of up to two months [11].

Similarly, a study by Yadav et al. investigated the properties of *E. aureum* for its role in nutrient removal in waste management and its potential role in the tertiary treatment of wastewater [12]. At this juncture, we are unsure of the mechanism of injury to the plants. Possibilities include direct toxicity of one or more ingredients, dehydration due to a change in water osmolality, or nutrient deficiency. We have not done any chemical analysis of the water or the plant parts.

It is therefore important to carefully examine the components of the toothpastes used in the study to understand the mechanism of injury to the plants. Camphor, an ingredient in toothpaste 1, has been known to exhibit phytotoxic effects. In a study conducted by Wang et al. involving Cinnamomum camphora extract, they found inhibition of seed germination and root elongation, nutrient absorption, and damage to the plasma membrane in the root [13]. Products with SLS and clove oil (Syzygium aromaticum) are known to have phytotoxic effects [14,15].

A study in 1996 by Heil et al. highlighted the possible genotoxicity of components of common dental care products such as eugenol and camphorquinone (CQ) [3]. C. camphora is not only an ingredient in toothpaste 1 used in this experiment but is also a commonly used ingredient in many other commonly used toothpaste and various consumer products such as cosmetics and personal care items. Furthermore, camphorquinone (CQ), a derivative of camphor, is a known cytotoxin causing oxidative stress and leading to CQ-induced DNA damage. [16] A study done by Volk et al. revealed that non-irradiated CQ is responsible for generating intracellular reactive oxygen species (ROS) leading to DNA damage and alteration, the consequences of which could be genotoxicity, mutagenesis, or carcinogenicity [17]. ROS are also known to further inhibit DNA repair mechanisms. The study stated that these results showed us that cells chronically exposed to low concentrations of CQ may ultimately accumulate DNA lesions over time.

Following this, many studies have been carried out to assess the toxicity of the various individual compounds commonly present in toothpaste. In a study done with human subjects, dentifrice with SLS caused the desquamation of epithelial cells of the oral mucosa in 60% of the subjects [18]. This study contributes to the literature on the ability of SLS to modify the barrier properties of human oral mucosa in vitro and in vivo [19,20].

Piper nigrum’s primary component is linalool, an oxygenated monoterpene [21]. Linalool, along with eucalyptol, has been identified as a major phytotoxic compound and is present in many essential oils, such as Zingiber officinale, along with eucalyptol [22]. Similarly, linalool and eugenol have both been known to induce hyperexcitation and epileptogenesis in snail neurons. [23,24] Multiple essential oils are known to contain compounds that are known to have phytotoxic effects, specifically oxygenated terpenes and monoterpenes [25]. The same are also being evaluated for their neurotoxic, genotoxic, embryotoxic, and allergenic properties and teratogenic effects [26]. Studies have shown the toxicity of Zingiber officinale in rats, resulting in liver and kidney damage on prolonged oral exposure, eventually leading to fatality [27].

The effects of excess fluoride have been studied in depth, and some of the well-known effects are DNA damage, oxidative stress, and apoptosis, leading to cell cycle changes in both oral mucosal cells and hepatocytes [28]. Fluoride is also known to cause dysplastic alterations in the oral soft tissues of Albino Wistar rats [29]. Results of another study showed that the percentages of apoptotic cells in the hippocampal region and pallium were significantly higher in rats exposed to fluoride [30]. Literature on the neurotoxic effects of fluoride has also further reported that, apart from causing cytotoxicity and genotoxicity, it can induce abnormal anxiety and depression-like symptoms in rats on account of dysregulated serotonin neurotransmission [31]. This is a very vital point to note in light of the increasing diagnoses of neuropsychiatric conditions in the global population.

Saccharin is a compound present in all toothpastes used in the study. While it was delisted as a possible carcinogen in the early 2000s, it is known to increase the risk of bladder cancer and hepatic dysfunction in rats on account of elevated oxidative stress in the liver [32].

The effects of oral consumption of silica, another ‌compound common in all toothpastes, were studied by Ogawa et al. [33]. The study found that, though silicon nanoparticles are considered non-toxic and are therefore approved for consumption, they have the capacity to aggravate intestinal inflammation. Their unfavorable effects on human health and condition therefore need to be taken into account in the light of their widespread and increasing consumption as a common ingredient as a preservative and thickening agent.

Titanium dioxide, present in toothpastes 2 and 3, has been considered unsafe for human consumption by the European Food Safety Authority (EFSA) since the year 2021, given its genotoxic effects [34]. Numerous studies, including those by Younes et al. and Chen et al., both indicated DNA breakage and chromosomal damage [34, 35]. An important highlight, however, remains the study by Shabbir et al. that the genotoxicity of titanium dioxide depends not only on particle size and other commonly studied mechanisms but also on the duration of exposure as well [36]. This is very pertinent in light of our study, given the duration of exposure of these toxic compounds to human cells and their possible adverse effects.

Sodium benzoate along with benzyl alcohol derivatives are compounds present in all three toothpastes. While these compounds are commonly used as preservatives in a variety of cosmetic products, there are certain studies that highlight the toxic effects of these substances. A study by Gaur et al. demonstrated the sodium benzoate-induced developmental abnormalities in animal models of zebrafish larvae, accompanied by oxidative stress and anxiety-like behavior [37]. The results were observed despite the study using lower concentrations of sodium benzoate than in other studies, highlighting the need for further investigation into the role of sodium benzoate as a common preservative. Sodium benzoate was similarly studied in mouse models for its effect on oxidative stress in the brain and subsequently was found to induce memory impairment with the potential to adversely impact the vulnerable neurological systems in children [38].

Previous studies have shown the time- and concentration-dependent toxicity of toothpaste ingredients on human fibroblast cells. [39] Ingredients in toothpastes like fluoride inhibit sodium-potassium ATPase and have been implicated in various systemic diseases [40,41]. As it is unknown which of these ingredients are toxic, we urge public health and toxicology departments worldwide to study the long-term consequences of toothpaste use, a daily activity that begins at the age of 2 and continues through the lifetime. Therefore, this research must be carried out urgently with further large-scale research in plants, cell lines, organoids, and animals along with longitudinal human studies. The slow toxicity of ingredients in toothpaste may explain the origin of many diseases like cancer, degenerative diseases, neurological disorders, psychiatric disorders, and childhood development disorders like attention deficit hyperactivity disorder (ADHD) and autism spectrum disorder (ASD).

This study, while important, is sure to raise some pertinent questions with regard to its relevance in humans. It is true that none of the compounds discussed above are being ingested and absorbed through the stomach and intestines. However, it must be taken into account that buccal absorption through mucosal membranes is a fast and effective process. Sublingually administered medication has garnered great focus for its accessibility and rapid absorption through the buccal mucosa [42]. The mucosal membrane of the oral cavity allows for both intercellular and intracellular absorption owing to its direct access to the systemic circulation through the internal jugular vein.

A recent study by Cohn et al. has shed light on the toxic effects of compounds found in commonly used substances, such as quaternary compounds found in disinfectants, softeners, and conditioners, and organophosphate flame retardants, present in furniture and electronics [43]. They were respectively found to be cytotoxic to developing oligodendrocytes and inhibiting oligodendrocyte development in human organoids. This holds severe and potent implications for research into neurodevelopmental and neuropsychiatric disorders as well as demyelinating conditions such as multiple sclerosis.

The strength of this study lies in the choice of money plants known for their high resilience and ability to survive in nutrient-deficient environments and provides a simple and robust model for assessing the toxicity of toothpaste ingredients. However, there are limitations to this study, which include a small sample size, not accounting for natural variability in plant responses with replicates, and not performing blinding and randomization. Larger studies must be done in the future with a larger number of plants in a randomized and blinded fashion to corroborate our findings and address these limitations. Also, it is unclear if the changes observed in the experimental conditions were due to toxicity, dehydration, or changes in osmolarity due to the sugars in the toothpaste. We were unable to analyze direct toxicity at the cellular and molecular levels due to the lack of facilities at our center. Detailed studies need to be carried out in well-equilibrated laboratories to evaluate the exact mechanisms leading to the results seen in this study. We hope that presenting these preliminary results will encourage further research, as this study raises an important public health concern.

## Conclusions

In this experiment involving toothpaste and plants, we demonstrated that the ingredients in toothpaste can be toxic to the survival of the money plant (*Epipremnum aureum*), a species known for its high resilience and survival capacity. Although we could not elucidate the mechanism of this injury in the current experiment, future studies are needed to investigate the mechanism of phytotoxicity caused by toothpaste ingredients at the cellular and molecular levels. We hope that these observations will inspire further research into the toxicity of toothpaste ingredients in various living organisms, including animals and humans. These findings raise important public health concerns, as toothpaste is used by most people worldwide.
